# Innoculation from Soap

**Published:** 1875-09

**Authors:** 


### INNOCULATION FROM SOAP.

Many years ago a great cry was raised in France on account of the innoculation of a person with a terrible disease, from the use of a toilet soap made by a famous perfumer. As is usual there, an investigation was had, when it was proved that the manufacturer used fat from human corpses, obtained for a small sum from a hospital attendant. Cancerous, scrofulous and syphilitic bodies were alike fish to the unprincipled soap-boilers' net, and so the terrible poisons were conveyed, in the form of highly-scented cakes of toilet soap, into thousands of homes.

The question comes up, as to the propriety or necessity of using animal fats in the manufacture of soaps which are to be used for toilet and household purposes. We all know that the grease used by soap-makers is often vile, disgusting stuff, rendered from diseased and decomposed animals, reeking with corruption, redolent with all foul odors, and often alive with those horrible crawling things which emanate from decaying animal substances. We would not touch with our hands the fearful mess for which the soap-maker willingly pays his money, for worlds. Yet we ignorantly rub it upon our bodies and luxuriate in the contact. True, it does not come to us as purulent and horrible pus, as filth and corruption, but as cleanly, sweet-scented soap, with some balmy, spicy, fragrant, floral name. What wonder that skin-diseases and irritations and scrofulas and cankers and blood poisons come from sources unknown, to polute the fountains of life and hasten the end?

We do not believe that decomposed animal fats, if indeed, any animal fats, ever should or ever need be used as a basis for soap. There are vegetable oils in abundance, and these are not subject to decomposition. They breed no pestilence and generate no worms. They become stale and rancid, but they give off no horrible odors, and their touch innoculates no one with disease.

The soap-maker will aver that the process of soap-making will disinfect and deodorise even the worst fats. This is true only in part. The admixture with alkali does in a great degree destroy the offensive odor, but it does by no means destroy disease [illegible]erms. Heat is a more potent agent t[illegible]is; yet, heat of the kind and degree u[illegible]d by the soap-maker, is not adequate to [illegible] Besides, a great deal of soap is made by the cold process, and in this all that is origin[illegible]y bad, remains.

Let us ceas[illegible] to buy soaps made from animal fats, and seek for those made from sweet, pure vegetable oils. These are abundant and cheap. The soap made from them is sweeter, more acceptable, more emollient, more healing and more fragrant. In seeking to avoid all avoidable sources of disease, let us see to it that the fiend does not appear in the form of the highly-perfumed cakes upon our wash-stands and toilet tables.

---

The consideration of the horrors of soap-poisoning brings to mind the excellent qualities of the tar-soap made by Packer. In this, we are assured, the only oil used is a pure and sweet vegetable product, which forms a plentiful lather, and leaves upon the skin a sense of smoothness and softness. In this soap there appears to be no free alkali—the alkaline re-action being absent on the application of tests. Probably tar alone, without other oily substance, could not be converted into a solid soap; but this soap of Packer's appears to approximate this condition very nearly. It cleanses and sweetens, and deodorises and purifies remarkably, and, in sores and eruptions and pimples and disfigurations of this kind, it possesses healing and soothing qualities peculiar to itself. It seems destined to take the place of the many questionable toilet soaps in our market.

